# GPT-4 generated answer rationales to multiple choice assessment questions in undergraduate medical education

**DOI:** 10.1186/s12909-025-06862-z

**Published:** 2025-03-04

**Authors:** Peter Y. Ch’en, Wesley Day, Ryan C. Pekson, Juan Barrientos, William B. Burton, Allison B. Ludwig, Sunit P. Jariwala, Todd Cassese

**Affiliations:** 1 Albert Einstein College of Medicine, Bronx, NY USA; 2 Department of Medicine, Albert Einstein College of Medicine, 1300 Morris Park Avenue, Bronx, NY USA

**Keywords:** ChatGPT, Artificial intelligence, Answer rationales, GPT-4, Clinical vignettes, Pre-clerkship assessments, LLM

## Abstract

**Background:**

Pre-clerkship medical students benefit from practice questions that provide rationales for answer choices. Creating these rationales is a time-intensive endeavor. Therefore, not all practice multiple choice questions (MCQ) have corresponding explanations to aid learning. The authors examined artificial intelligence’s (AI) potential to create high-quality answer rationales for clinical vignette-style MCQs.

**Methods:**

The authors conducted a single-center pre-post intervention survey study in August 2023 assessing 8 pre-clerkship course director (CD) attitudes towards GPT-4 generated answer rationales to clinical vignette style MCQs. Ten MCQs from each course’s question bank were selected and input into GPT-4 with instructions to select the best answer and generate rationales for each answer choice. CDs were provided their unmodified GPT-4 interactions to assess the accuracy, clarity, appropriateness, and likelihood of implementation of the rationales. CDs were asked about time spent reviewing and making necessary modifications, satisfaction, and receptiveness in using GPT-4 for this purpose.

**Results:**

GPT-4 correctly answered 75/80 (93.8%) questions on the first attempt. CDs were receptive to using GPT-4 for rationale generation and all were satisfied with the generated rationales. CDs determined that the majority of rationales were very accurate (77.5%), very clear (83.8%) and very appropriate (93.8%). Most rationales could be implemented with little or no modification (88.3%). All CDs would implement AI-generated answer rationales with CD editorial insights. Most CDs (75%) took ≤ 4 min to review a set of generated rationales for a question.

**Conclusion:**

GPT-4 is an acceptable and feasible tool for generating accurate, clear and appropriate answer rationales for MCQs in medical education. Future studies should examine students’ feedback to generated rationales and further explore generating rationales for question with media. The authors plan to explore the implementation of this technological application at their medical school, including logistics and training to create a streamlined process that benefits both learners and educators.

**Clinical trial:**

Not applicable; not a clinical trial.

**Supplementary Information:**

The online version contains supplementary material available at 10.1186/s12909-025-06862-z.

## Background

Medical student performance on United States Medical Licensing Exams (USMLE) is positively associated with the number of practice questions they complete and review before the administration of the exam [1–4]. This finding suggests that the formative feedback students glean from taking practice assessments, known as question response feedback, aids in subsequent exam performance. Question response feedback ranges in the amount of guidance provided to learners, from simply indicating whether the learner answered correctly (without providing any explanation), to providing only the correct answer explanation, to providing explanations for all answer responses. Providing exam item rationales for both correct and incorrect answer choices was associated with a statistically significant increase in score on a re-test when compared with only providing learners with the correct answer without further explanation [2]. While providing more extensive rationales aids learning, creating them is time-intensive for busy medical educators, many of whom may be practicing clinicians [3]. Therefore, many existing faculty-authored practice multiple choice questions (MCQ) provided to students to prepare for exams do not have corresponding answer explanations to aid student learning.

While artificial intelligence (AI) is a broad field focused on creating systems capable of mimicking human intelligence, large language models (LLMs) are a specific subset of AI, particularly within generative AI, designed to process and generate human-like text based on extensive language data. LLMs such as ChatGPT have been successfully used to answer USMLE-style MCQs and write MCQ assessments [5–8]. LLMs may also be capable of generating more extensive rationales for correct/incorrect responses for formative multiple-choice questions. However, the use of these tools may be met with some hesitation in academia due to perceived inaccuracy of explanations and occasional false outputs due to LLM extrapolations of data termed “hallucinations” [9]. Nevertheless, in medical education, AI, such as LLMs, seems to have a promising role as an assistive tool, albeit one that still likely requires human verification.

This study sought to evaluate whether GPT-4, the latest version of ChatGPT at the time of writing, could create high-quality extensive rationales for correct/incorrect answer responses to formative clinical vignette-style MCQs used in our institution’s pre-clerkship courses. We isolated clinical vignette-style MCQs since they represent the style of question presented on the USMLE exams that all medical students take. Additionally, we sought to understand pre-clerkship course director (CDs) perspectives and openness to using ChatGPT in their courses and whether CDs would view the use of AI-generated extensive rationales as an efficient use of their time.

Herein, we provide a proof of concept of ChatGPT’s ability to create high-quality extensive correct and incorrect answer rationales for formative clinical vignette-style MCQs used in our institution’s pre-clerkship courses with minor editorial input from CDs. We also provide evidence that CDs were pleased with the results, more likely to include extensive rationales for their questions, and were more likely to use ChatGPT for educational assistance after the trial. To our knowledge, this is the first study to comprehensively evaluate the use of GPT-4 for the purpose of extensive answer rationale generation in medical education and presents yet another exciting potential use of AI within medical education.

## Methods

We received an institutional review board exemption (Einstein IRB #2023–15126) to perform a pre-post intervention survey assessing pre-clerkship CD attitudes towards GPT-4 generated extensive answer rationales to in-house clinical vignette-style MCQs.

All organ system pre-clerkship CDs (*n* = 9) from a single US medical school were invited to participate by email following virtual recruitment at a CD committee presentation in August 2023. Eight CDs provided consent and completed an anonymous online pre-intervention survey (Appendix A) created via Microsoft Forms (Microsoft Corporation, Redmond, WA) regarding perceptions of and attitudes toward GPT-aided MCQ rationales. This survey also assessed CD perceived barriers to extensive rationale generation, with a particular focus on time spent writing rationales. Each respondent generated their own unique identifier so responses for the pre- and post-survey could be matched by the investigators while maintaining anonymity.

Formative MCQs administered to students in the 2021-22 academic year for each participating course were provided to the authors by the CDs in August 2023. Since MCQs in each course were heterogenous in formatting (ex. discrete questions, picture-based questions, clinical vignette questions), the authors filtered questions manually to identify only text-based clinical vignette-style questions.

Ten text-only clinical vignette-style MCQs from each course written by their respective CDs (total = 80) were selected at random after the manual filtering process and input into GPT-4 with standardized instructions to select the best answer and generate rationales for each answer option (prompts available in Appendix B). In cases where GPT-4 selected the wrong answer, it was given a second standardized prompt with the correct answer and instructions to re-generate rationales (Appendix B).

CDs were provided their 10 unmodified GPT-4 interactions in a formatted document along with the post-intervention survey (Appendix C) to assess the accuracy, clarity, appropriateness, and likelihood of implementation of the rationales and on a Likert scale (1–4), with 4 being the most positive sentiment for each category. CDs were also retrospectively asked about the average time spent reviewing and making necessary modifications for each answer rationale, their overall satisfaction with the rationales, and their overall receptiveness to using ChatGPT for this purpose.

### Data analysis

Pre- and post-survey results were anonymously matched. Descriptive statistics for demographics and other items in both the pre- and post-survey were generated for all participant responses. For pre-intervention survey variables that involved ranking (ex. most impactful to least impactful barriers), a frequency table was used to calculate scores for each option in a ranked-choice question. The scores were computed by multiplying the number of participants who selected each option by its weight, which was inversely proportional to the rank. The total score for each question was obtained by summing these weighted scores.

Dependent paired samples t-test was used to assess differences in receptiveness to using chat-based LLMs between matched pre- and post-intervention survey responses; ranked categorical choices were coded to their corresponding numerical Likert scale weights. All statistical analyses and generation of figures were conducted using SPSS version 29.0 for Mac (IBM, Armonk, NY) and GraphPad Prism version 10.1.1 for Mac (GraphPad Software, San Diego, CA).

## Results

Of the eight CDs surveyed, seven (87.5%) held an MD degree and one (12.5%) held a PhD degree (Table 1). All eight CDs agreed that providing rationales for both correct and incorrect responses was important for student learning, with five (62.5%) designating it as “very important.” Three (37.5%) CDs reported that they did not have rationales for any pre-existing MCQs in their current pre-clerkship course, with five others (62.5%) indicating they did not have rationales for the majority of practice items. Six (75.0%) CDs cited time expenditure as a barrier to writing rationales. When ranked by impact, time was the highest-ranked barrier to writing rationales. Of the five (62.5%) CDs who had previously written answer rationales, they estimated it took 10–30 + minutes to write answer rationales for a single MCQ on their own, with “concise writing” and “verifying accuracy” ranked as taking the most amount of time. In the pre-survey, six (75.0%) CDs were confident GPT-4 could generate implementable rationales while two (25.0%) were not confident. However, most CDs (87.5%) did not think GPT-4 could generate rationales without additional modification. Seven (87.5%) CDs were receptive to using GPT-4 for rationale generation, with three (37.5%) being “very receptive” prior to the intervention.

**Table 1:** Pre-intervention survey results

Variable	Count (%) [*n* = 8]
*Age (years)*	
35–44	4 (50)
45–54	3 (37.5)
65+	1 (12.5)
*Degree*	
MD	7 (87.5)
PhD	1 (12.5)
*What percentage of practice questions do you estimate you provide answer rationales for?*	
0%	3 (37.5)
1–20%	1 (12.5)
21–40%	1 (12.5)
41–60%	1 (12.5)
81–100%	1 (12.5)
Unknown	1 (12.5)
Rank potential barriers to writing rationales for correct/incorrect answer choices.*	
Time	29 points
Complexity of explaining correct/incorrect choices	20 points
Lack of administrative support	20 points
Answers are self-explanatory	13 points
Lack of understanding of technology	8 points
*Importance of providing rationale for both correct and incorrect responses*	
Somewhat important	3 (37.5)
Very important	5 (62.5)
*Estimated time to write an answer rationale for a formative MCQ*	
10–14 min	1 (12.5)
15–19 min	1 (12.5)
>=30 min	2 (25)
N/A; do not write answer rationales	4 (50)
Rank elements that take most to least amount of time in writing a rationale*	
Ensuring rationale is clear & concise	9 points
Verifying answer rationale accuracy	7 points
Ensuring rationale is appropriate level	4 points
*Will ChatGPT be able to generate answer explanations without additional input from a course director?*	
No	7 (87.5)
Yes	1 (12.5)
*Confidence in ChatGPT to generate usable answer rationales for use with students*	
Somewhat not confident	2 (25)
Somewhat confident	5 (62.5)
Very confident	1 (12.5)
*Receptiveness to using ChatGPT for rationale generation*	
Somewhat not receptive	1 (12.5)
Somewhat receptive	4 (50)
Very receptive	3 (37.5)

*A frequency table was used to calculate scores for each option in a 5-choice question. The scores were computed by multiplying the number of participants who selected each option by its weight, which was inversely proportional to the rank. The total score for each question was obtained by summing these weighted scores

**Fig. 1:**
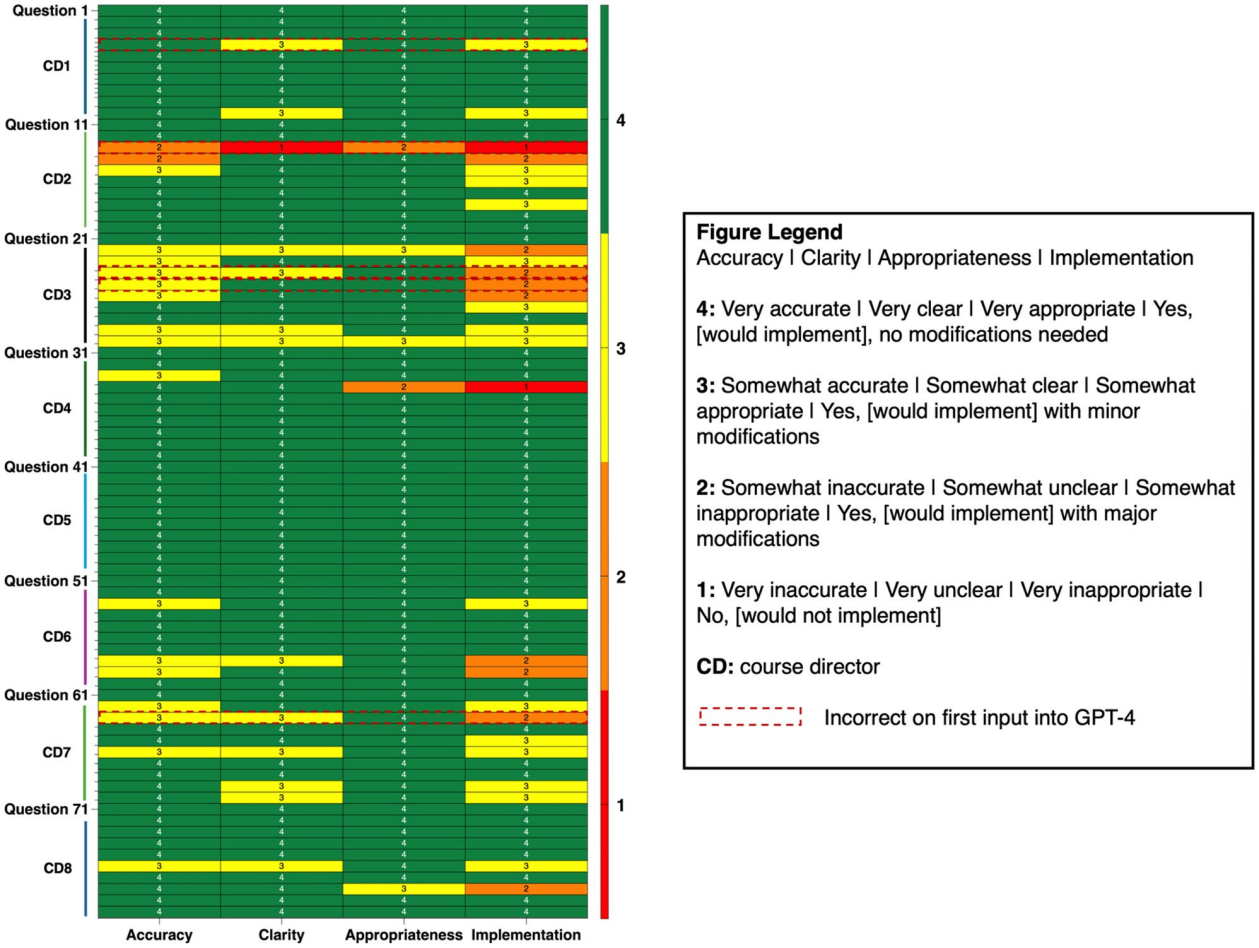
Post-intervention survey AI rationale evaluation heat map

Three selected questions with GPT-4 generated rationales (on first attempt: 2 correct, 1 incorrect) are available in Appendix D. GPT-4 correctly answered 75/80 (93.8%) questions on the first attempt. CDs evaluated each set of rationales per question for accuracy, clarity, appropriateness, and ability to be implemented with/without modifications (Table 2). CDs reviewed rationales for only the questions they had originally written. An overview of these evaluations per rationale set is detailed in Fig. 1. 78/80 of the rationales were deemed to be implementable. Of the two rationales that were determined to be not implementable even with edits, one of them was incorrectly answered by GPT-4 on the first attempt (but correct on the second attempt).

**Table 2:** Post-intervention survey results

Variable	Count (%)
*Evaluation of each generated rationale set per question* *(n = 80)*	
*Accuracy*	
Somewhat inaccurate	2 (2.5)
Somewhat accurate	16 (20)
Very accurate	62 (77.5)
*Clarity*	
Very unclear	1 (1.3)
Somewhat clear	12 (15)
Very clear	67 (83.8)
*Appropriateness*	
Somewhat inappropriate	2 (2.5)
Somewhat appropriate	3 (3.8)
Very appropriate	75 (93.8)
*Implementation*	
No	2 (2.5)
Yes, with major modifications	9 (11.3)
Yes, with minor modifications	16 (20)
Yes, no modifications needed	53 (66.3)
*Correct on first try*	75 (93.8)
***Course director evaluations*** *(n = 8)*	
*Would you implement chat-based AI generated answer rationales with no changes?*	
No	5 (62.5)
Yes	3 (37.5)
*Would you implement chat-based AI generated answer rationales with course director editorial insights?*	
Yes	8 (100)
*On average*,* how long did it take for you to review the rationale to answer choices per question generated by AI? (minutes)*	
> 0–2 min	2 (25)
3–4 min	4 (50)
5–9 min	2 (25)
*On average*,* estimate how long it would take for you to modify the rationale to answer choices per question generated by AI to the extent that it meets the level of quality and accuracy needed for implementation in your course (minutes)*	
> 0–2 min	1 (12.5)
3–4 min	2 (25)
5–9 min	3 (37.5)
10–14 min	2 (25)
*On average*,* how satisfied are you with the generated explanations?*	
Somewhat satisfied	1 (12.5)
Very satisfied	7 (87.5)
*Receptiveness to using chat-based AI to generate answer explanations in pre-clerkship course practice questions?*	
Somewhat receptive	3 (37.5)
Very receptive	5 (62.5)

In the post-intervention survey, all eight (100%) CDs noted that they would implement AI-generated answer rationales with CD editorial review, with three (37.5%) being comfortable with implementing with no further editorial changes among the rationales they reviewed. The majority of CDs (75.0%) reported taking an average of ≤ 4 min to initially review a set of generated rationales for a given question. Most (75.0%) reported an estimated average modification time of ≤ 9 min if a given question needed any changes to become usable for their own course. All CDs were satisfied with the generated explanations, with seven (87.5%) being “very satisfied.”

When comparing pre- and post-receptiveness to using LLMs for the generation of rationales, sentiment trended upwards (mean [SD], 3.25 [0.71], 3.63 [0.52], *p* =.197) with a medium effect size as measured by Cohen’s d = -0.504. Of the eight participating CDs, six (75.0%) pitched their own ideas of the use of AI in medical education, including using it to help format lectures, create case conference material, and help generate practice questions.

## Discussion

Overall, this study demonstrates the feasibility of using LLMs for the generation of answer rationales to pre-existing in-house pre-clerkship questions to enhance student learning. Our findings have several implications related to creating and providing rationales for both correct and incorrect answer options to MCQs and integrating AI in medical education. Pre-clerkship course directors clearly understand the importance of providing extensive rationales to aid student learning, but they are not routinely spending time on generating them because the task is time-consuming and labor-intensive. The barrier to creating extensive rationales hinders student learning from practice MCQs. Our results suggest that LLMs are a viable solution to overcoming this barrier and augmenting current pre-existing MCQs that do not have corresponding human-written answer rationales, thereby potentially improving student learning. The usefulness of LLMs in this context may go beyond pre-clerkship education and could also be relevant to clerkship and graduate medical training.

Furthermore, while our results indicate that GPT-4 is a promising tool for generating rationales, it is not a perfect one. Like other studies which explored the use of ChatGPT for generating USMLE-style MCQs, we also recommend that expert human oversight is still a vital component of this process [6, 8, 10]. While GPT-4 can produce accurate, clear, and appropriate rationales most of the time, it may not always capture the nuances of a particular question or meet the expectations and preferences of the faculty or students.

In this study, GPT-4 was only given the correct response if it initially provided an incorrect answer. We observed that the rationales generated for questions GPT-4 answered incorrectly on the first attempt were rated lower in quality by course directors compared to those where the correct answer was selected on the first try, even after GPT-4 was provided with the correct answer for a second attempt. Providing the correct answer in the initial input, rather than asking the LLM to first choose the correct answer then generate rationales, may assist the LLM in generating higher quality rationales and may be an area of future exploration. Another possibility that warrants further investigation is if the quality of these questions was lower than the others, such that more than one answer could be correct; if so, GPT-4 may be helpful in identifying lower quality questions needing improvement. Regardless, the per question evaluations suggest that human input is still necessary to ensure quality and accuracy in AI-generated rationales and implies that a collaborative approach between humans and AI is preferred.

Time was the most cited barrier for course directors to write rationales to MCQs. CDs estimated that it took no longer than 14 min on average to modify an AI-generated set of rationales for a given question, with most indicating it took between 5 and 9 min. While it is difficult to make definitive conclusions, half of the CDs who wrote their own answer rationales estimated it took more than 30 min to write a single answer rationale, suggesting that AI may save a substantial amount of time in this process.

An important finding of our study was that the pre-intervention skepticism of GPT-4 in the process of generating rationales was largely mitigated after the intervention, suggesting that exposure to AI-generated material in medical education may enhance faculty understanding and appreciation of its role and value, thus increasing the possibility of future adoption. Moreover, it may encourage faculty to explore and experiment with other AI applications and innovations to aid in their teaching and assessment practices.

## Conclusions

Positive feedback from CDs suggests that AI may play a valuable role in providing students with more high-quality answer rationales while reducing faculty time expenditure. Medical schools across the United States hold vast repositories of pre-existing course materials and MCQs that could be augmented with AI in this fashion. Garnering faculty support and familiarity with AI in these focused scenarios could lead to more seamless acceptance and widespread incorporation of AI in medical education in the future.

A limitation of this study is the lack of student feedback on the generated rationales. Future evaluation of these generated rationales by students is essential to verify that generated rationales truly provide utility while meeting their expectations of accuracy, clarity, and level-appropriateness. Student input can also be used to compare their perception of the quality of AI-generated versus human written rationales. Furthermore, this study was limited to text-only MCQs. The processing and generation of rich media like images have now become more accessible in mainstream consumer products like ChatGPT and should be evaluated in future studies. Finally, as the current study only drew upon resources from a single US MD school, these experiences with LLM incorporation for writing answer rationales to MCQs may not be generalizable to medical education in other countries.

Given the positive feedback from CDs, we will begin to explore the feasibility of real-world implementation of this technology in our medical school, including logistics and training to create a streamlined, sustainable process that ultimately benefits both our learners and educators.

In conclusion, this study demonstrated the feasibility and acceptability of using GPT-4, a large language learning model, to generate answer rationales for pre-existing in-house MCQs in pre-clerkship courses. We showed that GPT-4 can produce accurate, clear, and appropriate rationales that may require human modification prior to implementation for students. The current study suggests that using GPT-4 in this manner may increase the receptiveness and confidence of faculty towards AI, and reduces the time and effort required for writing rationales. Ultimately, a collaborative approach between human expertise and AI is optimal for its integration in medical education.

## Electronic supplementary material

Below is the link to the electronic supplementary material.

Supplementary Material 1

Supplementary Material 2

Supplementary Material 3

## Data Availability

The datasets generated and/or analyzed during the current study are not publicly available due to individual question ownership by course directors but are potentially available from the corresponding author on reasonable request with the consent of course directors on a case-by-case basis.
