# Supplementary material for: GPT-4 generated answer rationales to multiple choice assessment questions in undergraduate medical education
Source: BMC Med Educ. 2025 Mar 4;25:333. doi: 10.1186/s12909-025-06862-z (PMC11877964; doi:10.1186/s12909-025-06862-z)
Supplement: Supplementary file 1 — Supplementary Material 1 [file 12909_2025_6862_MOESM1_ESM.docx]

**Appendix A:** Pre-intervention survey

**Appendix B.** AI-generated MCQ rationales workflow


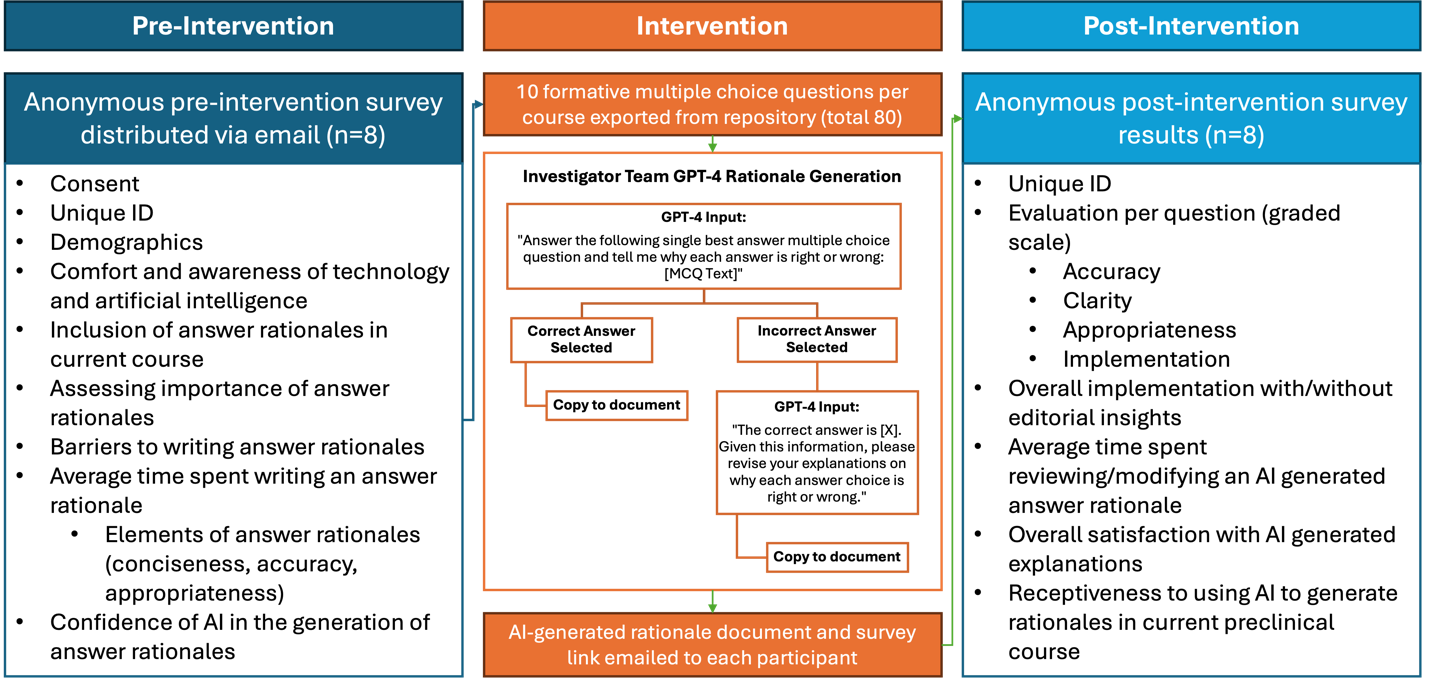


**Appendix C:** Post-intervention survey

**Appendix D:** Example question rationales

**CORRECT RATIONALE EXAMPLE 1 (RENAL):**

**INPUT:**

Answer the following single best answer multiple choice question and tell me why each answer is right or wrong:

“A 75-year-old woman is referred to you for evaluation of abnormal renal function. She has a history of HTN treated with medications for 32 years, diet-controlled DM for 4 years, and mild osteoarthritis. She is a retired telephone operator who is physically active and engaged in her medical care. She walks daily, follows a low sodium diet, and enjoys traveling with her friends. She has ongoing primary care. Her BP is controlled on three medications including lisinopril, hydrochlorothiazide, and amlodipine. Her physician has noted a slow rise in her serum creatinine for the past several years and recommended that she have a kidney function evaluation.
 
Her labs reveal a slow rise in serum creatinine from 0.6 mg/dl ten years ago to 1.2 mg/dl on her most recent labs.
Other labs include:
Glucose 105 mg/dl, HbA1c 6.9%
UA 1+ protein, no blood
24 hour urine total protein 200 mg
HBV negative, HCV negative, ANA positive 1:40 titer
 
A kidney biopsy is done:
Light: 6 normal appearing glomeruli, 4 globally sclerosed glomeruli, vascular intimal fibrosis and hyaline arteriolosclerosis, moderate tubular atrophy
IF: no staining
EM: one normal glomerulus with intact podocyte foot process structure
 
What is the cause of her stage 3 CKD?”

1. Hypertensive nephrosclerosis
2. HBV related membranous nephropathy
3. FSGS
4. Lupus nephritis
5. Diabetic nephropathy

**OUTPUT:**

Let's analyze each of the answer choices in light of the patient's presentation and kidney biopsy findings:

1**. Hypertensive nephrosclerosis**

   - The findings of vascular intimal fibrosis, hyaline arteriolosclerosis, and moderate tubular atrophy are consistent with hypertensive nephrosclerosis, especially given the history of long-standing hypertension. The globally sclerosed glomeruli are also consistent with this diagnosis. Chronic hypertension can cause damage to the renal blood vessels, leading to nephrosclerosis.

2.**HBV related membranous nephropathy**

   - The patient's HBV test is negative, making this etiology unlikely. Moreover, membranous nephropathy would typically show specific findings on immunofluorescence (IF) and electron microscopy (EM) that are not present in this patient's biopsy.

3. **FSGS (Focal Segmental Glomerulosclerosis)**

   - FSGS would show segmental scarring in some of the glomeruli. While there are some globally sclerosed glomeruli, the biopsy doesn't specifically indicate segmental scarring. Additionally, EM typically reveals podocyte foot process effacement in FSGS, but this patient's EM showed intact podocyte foot processes.

4**. Lupus nephritis**

   - Even though the patient has a low-titer positive ANA, the kidney biopsy findings are not typical of lupus nephritis. Lupus nephritis would generally show specific changes on light microscopy, immunofluorescence (deposits of immunoglobulins and/or complement), and electron microscopy (specific types of immune deposits), none of which are present here.

5.**Diabetic nephropathy**

   - Diabetic nephropathy usually presents with specific structural changes in the glomeruli called Kimmelstiel-Wilson nodules, increased mesangial matrix, and thickened glomerular basement membranes. The biopsy doesn't demonstrate these findings. Moreover, her diabetes has been present for only 4 years and appears to be relatively well-controlled (based on HbA1c).

Given the above analysis and the patient's clinical and biopsy findings, the most likely cause of her stage 3 chronic kidney disease (CKD) is:

**Hypertensive nephrosclerosis**

**CORRECT RATIONALE EXAMPLE 2 (ENDOCRINE):**

**INPUT:**

A 71-year-old male is referred for evaluation of bony pain. He reports insidious onset of thigh and hip pain. The pain is described as a deep, aching pain that has been present over the last few years. He denies any trauma or history of fracture. Review of laboratory data shows a normal serum vitamin D level, calcium and phosphorous and PTH. Alkaline phosphatase is elevated to 350 with an otherwise normal liver panel. X-rays of the patients hip and femur shows coarsening of the trabeculae and sclerosis.

What is the likely diagnosis in this patient? Explain the underlying pathophysiology of this disorder.

Excessive bone formation without resorption

Excessive osteoclast resorption with disorganized bone formation

Reduced bone mineralization

Reduced bone quality and quantity

**OUTPUT:**

The symptoms and findings described in this patient are consistent with Paget's disease of bone (or osteitis deformans). The most appropriate choice that corresponds to the pathophysiology of Paget's disease is:

****Excessive osteoclast resorption with disorganized bone formation****

Here's the breakdown of each answer choice:

1. ****Excessive bone formation without resorption**:** This is not consistent with Paget's disease. In Paget's, there is initially increased resorption of bone by osteoclasts, followed by a rapid and disorganized bone formation by osteoblasts. The new bone is weaker and larger, leading to pain and deformities.

2. ****Excessive osteoclast resorption with disorganized bone formation**:** This is the hallmark of Paget's disease. The disease often begins with excessive bone resorption, leading to an accelerated and abnormal bone formation. The newly formed bone is architecturally disorganized, which can cause it to be weak and prone to fractures. An elevated alkaline phosphatase is a marker of increased bone turnover, and coarsening of the trabeculae and sclerosis are radiographic findings consistent with the disease.

3. ****Reduced bone mineralization**:** This describes a process like osteomalacia or rickets, where there is a defect in the mineralization of the bone matrix. It is not the pathophysiology behind Paget's disease. The normal serum vitamin D, calcium, and phosphorus levels in this patient also make these conditions less likely.

4. ****Reduced bone quality and quantity**:** This is a general description that could apply to osteoporosis, where there's a reduction in bone mass or density and a deterioration of bone architecture. The patient's symptoms and laboratory/radiological findings are not consistent with osteoporosis.

In summary, the patient likely has Paget's disease of bone, which is characterized by excessive osteoclast resorption followed by disorganized bone formation. The exact cause of Paget's disease is still uncertain, but there is believed to be a combination of genetic and environmental factors. Some theories suggest a slow viral infection might trigger the disease in genetically susceptible individuals.

**INCORRECT RATIONALE EXAMPLE 1 (RENAL):**

**INPUT:**

Answer the following single best answer multiple choice question and tell me why each answer is right or wrong:

“A 24 year old woman presents to the ER with fatigue and is found to be hyponatremic. Her serum Na+ 124 meq/L. On physical exam she is thin and in no distress. Her BP is 105/62 with heart rate 66. She has no edema and her exam is unremarkable. She is given IV fluids and a regular diet. Her medical team is perplexed because her serum sodium does not improve after three days in the hospital. The medical student on the renal consult team sits down with the patient and obtains some important history that she had not previously shared with the team. She has a very stressful relationship with her domestic partner causing her to stop eating. She has a history of an eating disorder. However, she does her best to keep up with good fluid intake. She had thought that coming to the hospital would give her respite from her stress and anxiety but it has actually worsened because she is worried about her son. She has been throwing out the majority of the food on her tray when the nurses and patient care technicians are not looking but continues to drink all the liquids provided to her.
 
Which values are consistent with the cause of her hyponatremia?”

1. ADH = undetectable; Urine osmolality = 50 mosm/kg; Total body volume  = Normal
2. ADH = elevated; Urine osmolality = 1200 mosm/kg; Total body volume  = Low
3. ADH = elevated; Urine osmolality = 400 mosm/kg; Total body volume  = Normal
4. ADH = undetectable; Urine osmolality = 300 mosm/kg; Total body volume  = High
5. ADH = elevated; Urine osmolality = 900 mosm/kg; Total body volume  = High

**OUTPUT:**

This patient's presentation suggests the syndrome of inappropriate antidiuretic hormone secretion (SIADH). SIADH involves the inappropriate secretion of ADH, leading to water retention and hyponatremia. Factors that can trigger SIADH include stress, medications, malignancies, and other diseases. The history of stress, coupled with maintaining fluid intake despite poor food intake, makes SIADH a strong consideration.

Given the SIADH hypothesis, we'd expect:

- Elevated ADH (because it's inappropriately being secreted even when not needed).

- An elevated urine osmolality (because ADH causes the kidneys to reabsorb water, leading to a concentrated urine).

- Normal total body volume (because although the patient is retaining water, she's not necessarily "overloaded").

Let's examine the choices:

1. **ADH = undetectable; Urine osmolality = 50 mosm/kg; Total body volume = Norma**l: This doesn't fit. We'd expect elevated ADH and higher urine osmolality in SIADH.

2. **ADH = elevated; Urine osmolality = 1200 mosm/kg; Total body volume = Low:** The elevated ADH and high urine osmolality fit with SIADH. However, a low total body volume does not.

3. **ADH = elevated; Urine osmolality = 400 mosm/kg; Total body volume = Normal:**This option fits best with SIADH. Elevated ADH, increased urine concentration (though not as high as 1200 mosm/kg but still significantly elevated), and normal total body volume are consistent with the condition.

4.**ADH = undetectable; Urine osmolality = 300 mosm/kg; Total body volume = High:** The ADH value is inconsistent with SIADH, and the urine osmolality isn't as concentrated as we'd expect with elevated ADH.

5. **ADH = elevated; Urine osmolality = 900 mosm/kg; Total body volume = High:** Elevated ADH and high urine osmolality fit with SIADH, but the high total body volume does not.

The most consistent option with the patient's presentation and SIADH is:

**ADH = elevated; Urine osmolality = 400 mosm/kg; Total body volume = Normal.**

**INPUT:**

**The correct answer is 1. Given this information, please revise your explanations on why each answer choice is right or wrong.**

**OUTPUT:**

Of course, let's reassess the situation.

Given the presentation of the 24-year-old woman, the clinical picture suggests that she is drinking excessive amounts of water (polydipsia), especially in the context of poor food (and thus solute) intake. This can result in a dilutional hyponatremia. This scenario differs from SIADH in one major aspect: SIADH would have inappropriate retention of water due to elevated ADH, but in primary polydipsia (excessive drinking), the body would try to excrete the excess water, resulting in a low urine osmolality.

Let's re-evaluate the answer choices:

1. **ADH = undetectable; Urine osmolality = 50 mosm/kg; Total body volume = Normal**: This fits with the diagnosis of primary polydipsia. If she's consuming an excessive amount of water, the body will suppress ADH (since there's no need to retain water) and produce dilute urine with a low osmolality.
2. **ADH = elevated; Urine osmolality = 1200 mosm/kg; Total body volume = Low:**This would suggest SIADH or other causes of volume contraction, which doesn't fit her presentation.
3. **ADH = elevated; Urine osmolality = 400 mosm/kg; Total body volume = Normal:**This could also suggest SIADH but not as strongly as choice 2. Still, it doesn't fit primary polydipsia.
4. **ADH = undetectable; Urine osmolality = 300 mosm/kg; Total body volume = High:**While ADH being undetectable matches the scenario of excessive water intake, the urine osmolality is not low enough, and a high total body volume is not described in the vignette
5. **ADH = elevated; Urine osmolality = 900 mosm/kg; Total body volume = High:** This does not fit primary polydipsia or SIADH well and suggests other pathologies, like heart failure or renal issues.

Upon this reflection, the best match for a patient with primary polydipsia is:

**ADH = undetectable; Urine osmolality = 50 mosm/kg; Total body volume = Normal.**
