# Supplementary material for: GPT-4 generated answer rationales to multiple choice assessment questions in undergraduate medical education
Source: BMC Med Educ. 2025 Mar 4;25:333. doi: 10.1186/s12909-025-06862-z (PMC11877964; doi:10.1186/s12909-025-06862-z)
Supplement: Supplementary file 2 — Supplementary Material 2 [file 12909_2025_6862_MOESM2_ESM.pdf]

AI-assisted answer rationale generation in medical education pre-evaluation questionnaire

Thank you for agreeing to participate in our study on the use of AI in the generation of answer rationales for clinical vignette style questions!

Please fill out the following survey BEFORE you have read through any generated rationales that have been sent to you.

Thank you,
Artificial Intelligence in Medical Education Research Team

Peter Ch'en BS, Albert Einstein College of Medicine [peter.chen@einsteinmed.edu]
Wesley Day BS, Albert Einstein College of Medicine [wesley.day@einsteinmed.edu]
Ryan Pekson PhD, Albert Einstein College of Medicine [ryan.pekson@einsteinmed.edu]
Juan Barrientos MA, MS, Albert Einstein College of Medicine
[juan.barrientos@einsteinmed.edu]
William Burton, PhD, Albert Einstein College of Medicine [william.burton@einsteinmed.edu]

* Required

Consent

Background/Significance:
We are doing a research study that aims to generate a proof-of-concept for the role of chat-based AI in assisting generation of answer choice rationales for medical education assessment ques-

tions. We would like you to participate in this study. You are receiving this email because you are a faculty member directing a pre-clerkship course at Einstein and is actively involved in creating multiple-choice items both for formative and summative assessments.

Study Design:
If you agree, we will ask you to complete 2 questionnaires, both of which will be sent to you by email – one pre-evaluation questionnaire, and one post-evaluation questionnaire (after receiving the generated answer rationales for your review). We anticipate it will take ~ 5 – 10 minutes to complete each survey. Your responses will be deidentified and tagged with a unique identifier.

If you feel uncomfortable with answering certain questions, please know that you do not have to answer any question and you may stop completing the survey at any time.

Study Population:
Course directors of any pre-clerkship course at Einstein are eligible to be included in the study and complete this survey.

Risk/Benefit:
There is no risk to completing or not completing the survey. One potential risk of taking part in this study is the possibility of a loss of confidentiality or privacy. Loss of privacy means having your personal information shared with someone who is not on the study team and was not supposed to see or know about your information. The study team and survey do not ask for any personally identifiable information, and we encourage you not to enter any in the survey. However, if you do enter any personally identifying information, we still plan to protect your privacy. No identifying data will be downloaded to personal computers or workstations. They will be stored on HIPAA-compliant, secure Einstein/Montefiore cloud databases. We do not plan to share the information from this study with other researchers. Your study information will be kept as long as it is useful for this research.

There is a potential benefit in the process and conclusions of the study as it may empower medical education faculty members with the ability to engage AI to help generate answer choice rationales, which could potentially improve both their course and student learning. Furthermore, the generated answer rationales may augment your teaching.

The researchers and study staff follow federal and state laws to protect your privacy. This part of the consent form tells you what information we collect that may be shared in the dissemination of the findings of this research study. If you do not provide your consent, you may not participate in the research.

The purposes of these uses and disclosures are to (1) conduct the study and (2) make sure the study is being done correctly.

Information from this study may be used in future research studies by our study team.

The people who can see your research records includes:
- The research team and staff who work with them
- Groups that review research such as the Einstein IRB, and the Office for Human Research Protections

All of these groups are required to keep information collected in this study confidential.

If you change your mind and don't want your information used for the study anymore, you can call Peter Ch'en at 425-324-6443 or call the Einstein IRB at 718-430-2253. If the information collected in the study has already been used, it cannot be cancelled.

Completing this questionnaire indicates your consent to participate in this study.

Background Information

1. Unique Identifier *

Please create a unique identifier by typing in the first 3 letters of the city you were born in and the last four digits of your MOBILE phone number.

For example, if you were born in the Bronx, NY and your phone number is 123-456-7890, your identifier would be BRO7890.

This identifier is private and will only be used as a way to match your responses from the pre- to post-survey.

2. Age (years)

- ◯ 25-34
- ◯ 35-44
- ◯ 45-54
- ◯ 55-64
- ◯ 65+

3. Choose all degrees that apply

- ☐ MD
- ☐ PhD
- ☐ EdD
- ☐ MBA
- ☐ MPH
- ☐ MHPE
- ☐ Other

4. Number of years as a course director at Einstein

- ○ ≤3 years
- ○ >3 or ≤ 6 years
- ○ >6 or ≤ 10 years
- ○ >10 years

5. Please rate your comfort with using technology in general

- Not comfortable at all
- Somewhat not comfortable
- Somewhat comfortable
- Very comfortable

6. Please rate your awareness of chat-based artificial intelligence (AI)

- Not aware at all
- Somewhat not aware
- Somewhat aware
- Very aware

7. Please rate your comfort level with using chat-based artificial intelligence (AI)

- ◯ Not comfortable at all
- ◯ Somewhat not comfortable
- ◯ Somewhat comfortable
- ◯ Very comfortable

8. Have you tried ChatGPT or a related chat-based AI service (ex. Google Bard, Bing Chat, etc.) yet?

- ◯ Yes
- ◯ No

9. How long do you estimate you have spent on chat-based AI in total?

- 0 minutes
- > 0-9 minutes
- 10-29 minutes
- 30 ≤ 60 minutes
- > 1-2 ≤ hours
- > 2-4 ≤ hours
- > 4-8 ≤ hours
- > 8-12 ≤ hours
- > 12-18 ≤ hours
- > 18-24 ≤ hours
- Other

Study Questions

We are interested in the impact that chat-based AI can have in crafting accurate and comprehensive answer rationales for formative practice multiple choice questions provided by course directors.

10. Do you include correct and incorrect answer rationale/explanations with your formative practice questions on Canvas? Please check all that apply.

- [ ] Yes, provide rationales for **correct** answers
- [ ] Yes, provide rationales for **incorrect** answers
- [ ] Do not provide rationales at all (neither)
- [ ] Other

11. What percentage of practice questions do you estimate you provide answer rationales for?

- ◯ 0%
- ◯ 1-20%
- ◯ 21-40%
- ◯ 41-60%
- ◯ 61-80%
- ◯ 81-100%
- ◯ Other

12. The following are potential barriers to writing rationales for correct/incorrect answer choices on practice multiple choice questions. Please check all that apply.

- ☐ Time
- ☐ Complexity of explaining correct/incorrect choices
- ☐ Rationale not needed as answers are self-explanatory
- ☐ Lack of administrative support
- ☐ Lack of understanding of Canvas or other technology
- ☐ Other

13. The following are potential barriers to writing rationales for correct/incorrect answer choices on practice multiple choice questions.

    Please rank the following from biggest to smallest barrier for you.

    - Lack of understanding of Canvas or other technology
    - Lack of administrative support
    - Rationale not needed as answers are self-explanatory
    - Time
    - Complexity of explaining correct/incorrect choices

14. What other barriers do you anticipate? Please list them here along with where it would be placed in the ranking above. Otherwise, write N/A.

15. Please rate the importance of the following three items.

	Not at all important	Somewhat unimportant	Somewhat important	Very important
Importance of providing rationale for **correct** choices	◯	◯	◯	◯
Importance of providing rationale for **incorrect** choices	◯	◯	◯	◯
Importance of providing rationale for **both correct and incorrect** responses to an MCQ	◯	◯	◯	◯

16. On average, estimate how long it would typically take you, in minutes, to generate answer rationale(s) for a **single formative multiple choice question** on Canvas (if applicable).

    If you do not write answer rationales for students to see, please indicate "N/A" below.

- ◯ 1-2 minutes
- ◯ 3-4 minutes
- ◯ 5-9 minutes
- ◯ 10-14 minutes
- ◯ 15-19 minutes
- ◯ 20-24 minutes
- ◯ 25-29 minutes
- ◯ ≥30 minutes
- ◯ N/A; do not write answer rationales
- ◯ Other

17. In your process of writing answer rationales for MCQs, rank what takes the most to least amount of time.

- Ensuring rationale is clear and concise
- Ensuring rationale is appropriate to the level of the learner
- Verifying answer rationale accuracy

18. If you have an answer that you would like to add to the ranking above, please list it here along with where it would be placed in the ranking. Otherwise, write N/A.

19. Do you think chat-based AI like ChatGPT can generate answer explanations to clinical vignette style questions that do not require any additional input from a course director? *

- Yes
- No

20. How confident do you feel AI such as ChatGPT can generate answer explanations/rationale that you could use with students?

- ◯ Very not confident
- ◯ Somewhat not confident
- ◯ Somewhat confident
- ◯ Very confident

21. What concerns do you have for using ChatGPT for this purpose?

22. How receptive are you to using chat AI to generate answer explanations for your preclinical course practice questions?

- ◯ Very not receptive
- ◯ Somewhat not receptive
- ◯ Somewhat receptive
- ◯ Very receptive

23. Any additional comments or thoughts to add?

This content is neither created nor endorsed by Microsoft. The data you submit will be sent to the form owner.

Microsoft Forms
