# Supplementary material for: GPT-4 generated answer rationales to multiple choice assessment questions in undergraduate medical education
Source: BMC Med Educ. 2025 Mar 4;25:333. doi: 10.1186/s12909-025-06862-z (PMC11877964; doi:10.1186/s12909-025-06862-z)
Supplement: Supplementary file 3 — Supplementary Material 3 [file 12909_2025_6862_MOESM3_ESM.pdf]

AI-assisted answer rationale generation in medical education post-evaluation questionnaire

Thank you for agreeing to participate in our study on the use of AI in the generation of answer rationales for clinical vignette style questions!

Please fill out the following survey AFTER you have read through and edited (if needed) the generated rationales that have been sent to you.

* Required

Evaluation per question

Question 1 corresponds with Question 1 on the Word document you received with the AI-generated answer rationales

1. Unique Identifier *

Please enter your unique identifier by typing in the first 3 letters of the city you were born in and the last four digits of your MOBILE phone number. **This is the same identifier you used for the pre-survey.**

For example, if you were born in the Bronx, NY and your phone number is 123-456-7890, your identifier would be BRO7890.

This identifier is private and will only be used as a way to match your responses from the pre- to post-survey.

2. Accuracy

How factually correct were each of the answer rationales?

	Very inaccurate	Somewhat inaccurate	Somewhat accurate	Very accurate
Question 1	○	○	○	○
Question 2	○	○	○	○
Question 3	○	○	○	○
Question 4	○	○	○	○
Question 5	○	○	○	○
Question 6	○	○	○	○
Question 7	○	○	○	○
Question 8	○	○	○	○
Question 9	○	○	○	○
Question 10	○	○	○	○

3. Clarity

How easy to follow were each of the answer rationales?

	Very unclear	Somewhat unclear	Somewhat clear	Very clear
Question 1	○	○	○	○
Question 2	○	○	○	○
Question 3	○	○	○	○
Question 4	○	○	○	○
Question 5	○	○	○	○
Question 6	○	○	○	○
Question 7	○	○	○	○
Question 8	○	○	○	○
Question 9	○	○	○	○
Question 10	○	○	○	○

4. Appropriateness

Was the medical terminology and concepts used in answer rationales appropriate for the level of the learner?

	Very inappropria te	Somewhat inappropria te	Somewhat appropriate	Very appropriate
Question 1	○	○	○	○
Question 2	○	○	○	○
Question 3	○	○	○	○
Question 4	○	○	○	○
Question 5	○	○	○	○
Question 6	○	○	○	○
Question 7	○	○	○	○
Question 8	○	○	○	○
Question 9	○	○	○	○
Question 10	○	○	○	○

5. Implementation

Would you implement this answer rationale into your course?

	No	Yes, with major modifications	Yes, with minor modifications	Yes, no modifications needed
Question 1	○	○	○	○
Question 2	○	○	○	○
Question 3	○	○	○	○
Question 4	○	○	○	○
Question 5	○	○	○	○
Question 6	○	○	○	○
Question 7	○	○	○	○
Question 8	○	○	○	○
Question 9	○	○	○	○
Question 10	○	○	○	○

6. If you chose "no" for any of the above, why not?

7. Please check any questions that were incorrect on the first attempt

If all questions were correct on the first attempt, skip this question

- [ ] Question 1
- [ ] Question 2
- [ ] Question 3
- [ ] Question 4
- [ ] Question 5
- [ ] Question 6
- [ ] Question 7
- [ ] Question 8
- [ ] Question 9
- [ ] Question 10

8. Would you implement chat-based AI generated answer rationales with no changes?

- ◯ Yes
- ◯ No
- ◯ Other

9. Would you implement chat-based AI generated answer rationales with course director editorial insights?

- ◯ Yes
- ◯ No
- ◯ Other

Post-evaluation questionnaire

10. On average, how long did it take for you to review the rationale to answer choices **per question** generated by AI? (minutes)

- 0 minutes (none)
- >0-2 minutes
- 3-4 minutes
- 5-9 minutes
- 10-14 minutes
- 15-19 minutes
- 20-24 minutes
- 25-29 minutes
- ≥30 minutes

11. On average, estimate how long it would take for you to modify the rationale to answer choices **per question** generated by AI to the extent that it meets the level of quality and accuracy needed for implementation in your course (minutes)

- ○ 0 minutes (none)
- ○ >0-2 minutes
- ○ 3-4 minutes
- ○ 5-9 minutes
- ○ 10-14 minutes
- ○ 15-19 minutes
- ○ 20-24 minutes
- ○ 25-29 minutes
- ○ ≥30 minutes

12. On average, how satisfied are you with the generated explanations?

- ◯ Very dissatisfied
- ◯ Somewhat dissatisfied
- ◯ Somewhat satisfied
- ◯ Very satisfied

13. Please elaborate on why you chose your above answer

14. After reviewing the generated answer rationales, how receptive are you to using chat-based AI to generate answer explanations for your preclinical course practice questions?

- ◯ Very not receptive
- ◯ Somewhat not receptive
- ◯ Somewhat receptive
- ◯ Very receptive

15. Please elaborate on your above answer, why or why not?

16. Would you like more faculty development on the use of AI for providing rationales?

- ○ Yes
- ○ No
- ○ Maybe
- ○ Other

17. Would you like more faculty development on other uses for AI?

- ○ Yes
- ○ No
- ○ Maybe
- ○ Other

18. What types of development would you like to see?

19. Are you interested in being involved in future studies of the usage of AI in medical education?

- ◯ Yes
- ◯ No
- ◯ Maybe
- ◯ Other

20. Any additional comments or thoughts to add?

This content is neither created nor endorsed by Microsoft. The data you submit will be sent to the form owner.

Microsoft Forms
